# Automated mediastinal lymph node detection from CT volumes based on intensity targeted radial structure tensor analysis

**DOI:** 10.1117/1.JMI.4.4.044502

**Published:** 2017-11-09

**Authors:** Hirohisa Oda, Kanwal K. Bhatia, Masahiro Oda, Takayuki Kitasaka, Shingo Iwano, Hirotoshi Homma, Hirotsugu Takabatake, Masaki Mori, Hiroshi Natori, Julia A. Schnabel, Kensaku Mori

**Affiliations:** a Nagoya University, Graduate School of Information Science, Furo-cho, Chikusa-ku, Nagoya, Japan; b King’s College London, Division of Imaging Sciences and Biomedical Engineering, St. Thomas’ Hospital, London, United Kingdom; c Nagoya University, Graduate School of Informatics, Furo-cho, Chikusa-ku, Nagoya, Japan; d Aichi Institute of Technology, School of Information Science, Yakusa-cho, Toyota, Japan; e Nagoya University Graduate School of Medicine, Showa-ku, Nagoya, Japan; f Sapporo-Kosei General Hospital, Chuo-ku, Sapporo, Japan; g Sapporo Minami-Sanjo Hospital, Chuo-ku, Sapporo, Japan; h Keiwakai Nishioka Hospital, Toyohira-ku, Sapporo, Japan

**Keywords:** computer-aided detection, lung cancer, local intensity structure analysis, structure tensor

## Abstract

This paper presents a local intensity structure analysis based on an intensity targeted radial structure tensor (ITRST) and the blob-like structure enhancement filter based on it (ITRST filter) for the mediastinal lymph node detection algorithm from chest computed tomography (CT) volumes. Although the filter based on radial structure tensor analysis (RST filter) based on conventional RST analysis can be utilized to detect lymph nodes, some lymph nodes adjacent to regions with extremely high or low intensities cannot be detected. Therefore, we propose the ITRST filter, which integrates the prior knowledge on detection target intensity range into the RST filter. Our lymph node detection algorithm consists of two steps: (1) obtaining candidate regions using the ITRST filter and (2) removing false positives (FPs) using the support vector machine classifier. We evaluated lymph node detection performance of the ITRST filter on 47 contrast-enhanced chest CT volumes and compared it with the RST and Hessian filters. The detection rate of the ITRST filter was 84.2% with 9.1 FPs/volume for lymph nodes whose short axis was at least 10 mm, which outperformed the RST and Hessian filters.

## 1 Introduction

Lung cancer is the leading cause of cancer-related deaths in the United States^1^ and China.^2^ It is also the leading cause of cancer-related deaths among men worldwide.^3^ There are several methods of treatment: surgery, chemotherapy, and radiotherapy. To choose the best treatment procedure, cancer staging based on the TNM staging system^4^ is required. Three factors are focused on in staging: T (tumor), N (lymph nodes), and distant M (metastasis). In the preoperative diagnosis of lung cancer, radiologists check mediastinal lymph nodes on computed tomography (CT) volumes. However, because lymph nodes are small and their silhouettes are not clear, they might be overlooked. To prevent medical doctors from overlooking them and to lighten their burden, a computer-aided detection system for automated lymph node detection is strongly desired.

There are various approaches for detecting lymph nodes from CT volumes: random forest statistical classifier,^5^ local intensity structure analyses based on Hessian matrix,^6^^–^^8^ or radial structure tensor (RST).^9^ Three-dimensional (3-D) Haar-like features are 3-D feature point detection algorithms that can detect blob-like structures in volumetric images. Barbu et al.^10^ introduced 3-D Haar-like features for axillary, pelvic, and abdominal lymph nodes. Feulner et al.^11^ utilized them for mediastinal lymph node detection. The random forest statistical classifier is a supervised machine learning technique that can be utilized for enhancing target objects in image volumes. Cherry et al.^12^ utilized random forest statistical classifiers for abdominal lymphadenopathy detection.

Local intensity structure analysis based on the Hessian matrix has been widely used for many algorithms of automated detection and segmentation of organs^13^^–^^15^ and lesions.^16^^–^^19^ The Hessian matrix is computed for each location and describes the local intensity structure as a blob, line, or sheet around the location, and whether it is brighter or darker than surrounding regions. The bright blob-like structure enhancement filter based on the Hessian matrix (Hessian filter) responds with a high value at the central part of the blob-like regions, which are brighter than surrounding regions. Feuerstein et al.^20^ proposed a mediastinal lymph node detection algorithm using this. Another algorithm proposed by Liu et al.^21^ is also based on Hessian analysis. Random forest^5^ and support vector machine (SVM)^22^ classifiers were introduced to improve performance. Roth et al.^23^ introduced deep convolutional neural networks^24^ for further improvement.

Another detection algorithm is through local intensity structure analysis based on RST.^9^ Nimura et al.^25^ introduced the bright blob-like structure enhancement filter based on RST (RST filter) for detecting the abdominal lymph nodes. Its benefit is that it can enhance the entire region of the target object, in contrast to the Hessian filter, which enhances only the central part of the region. The RST filter can capture the lymph node shape more properly than the Hessian filter. The features extracted can be used to determine whether each candidate region is a true positive (TP) or a false positive (FP) using machine learning techniques. However, the RST filters have so far not performed well on mediastinal lymph node detection. The current RST filter fails when tissues have largely varying intensity distributions close to the target, for instance, in the case of air and contrasting blood vessels.

Lymph nodes on CT volumes typically show the following characteristics:

1. slightly higher intensity than surrounding regions,
2. spherical shape,
3. narrow intensity range similar to soft tissue.

**Fig. 1:**
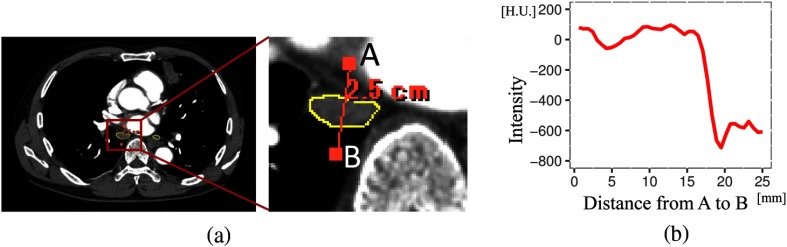
Intensity profile of lymph node. (a) Example of axial slice and its magnification of lymph node. Yellow represents lymph nodes. (b) Intensity profile on line segment A–B shown in (a).

To identify lymph nodes in CT images, the RST filter is designed to detect the regions with characteristics (1) and (2). However, mediastinal lymph node detection is a challenging problem in medical imaging because mediastinal lymph nodes are closely surrounded by many structures, such as contrast-enhanced blood vessels or air, as shown in Fig. 1. Although there are several works on lymph node detection on CT volumes, they fail to detect such lymph nodes. To overcome this problem, this paper proposes a new filter called the intensity targeted radial structure tensor (ITRST) filter, that is able to detect lymph nodes located around anatomical structures of extremely higher or lower intensities. The idea of the ITRST filter is to ignore extremely higher or lower intensity regions in RST computation to meet the requirement of characteristic (1). This allows us to detect lymph nodes neighboring regions with extremely high or low intensities.

The contribution of this paper is (a) to propose a new lymph node detection filter called the ITRST filter and (b) to evaluate its performance using artificial and clinical CT volumes.

In Sec. 2, we propose the automated mediastinal lymph node detection algorithm based on the ITRST filter and SVM classifier. In Sec. 3, we present two experiments showing the efficacy of the ITRST filter. The results are presented and discussed in subsequent sections.

## 2 ITRST-Based Lymph Node Detection Algorithm

### 2.1 ITRST Filter

This paper introduces a new blob-like structure enhancement filter called the ITRST filter, which is a modified version of the RST filter. Before explaining the ITRST filter, we will give a brief overview of the RST filter. The RST is given as the second-order tensor matrix (3×3 matrix) and is given by

$$T(x)=\underset{i}{\sum }\underset{j}{\sum }\alpha_{i,j}r_{i}g_{i,j}^{T},$$ (1)

where $x={\left(x,y,z\right)}^{T}$ is the location of a voxel where the RST is computed, $r_{i}$ denotes an i’th search direction from x, and $g_{i,j}$ is a local gradient vector of $I(x_{i,j})$. $\alpha_{i,j}$ is an opacity, given by

$$\alpha_{i,j}=\{\begin{array}{ll} 0 & \text{if}\;|I(x)-I(x_{i,j})|<t_{\min},\\ \frac{\left|I(x)-I(x_{i,j})\right|}{\left|t_{\max}-t_{\min}\right|} & \text{if}\;t_{\min}\leq |I(x)-I(x_{i,j})|<t_{\max},\\ 1 & \text{otherwise}, \end{array}$$ (2)

where i is the index of search directions, j is the index of search steps of each search, $x_{ij}$ is a voxel located in the j’th search step on the i’th search direction, $t_{\min}$ and $t_{\max}$ ($t_{\min}<t_{\max}$) are parameters for controlling the sensitivity of the gradient. When an accumulated opacity $\beta_{i}=\underset{j}{\sum }\alpha_{i,j}≃1$ or a search length becomes $t_{\mathrm{len}}$ or larger, a search for the i’th search direction is terminated.

Eigenvalues $\lambda_{0}$, $\lambda_{1}$, $\lambda_{2}(|\lambda_{0}|\geq |\lambda_{1}|\geq |\lambda_{2}|)$ of $T(x)+T^{T}(x)$ represent the magnitude of the gradient directing the corresponding eigenvector around x. If all eigenvalues are negative, I(x) is brighter than the surrounding region. The larger the magnitude of the eigenvalue, the larger the gradient. The eigenvalues are utilized to enhance the bright blob-like structure regions that have the condition $\lambda_{1}≃\lambda_{2}≃\lambda_{3}\ll 0$ using an evaluation formula. For example, a simple evaluation formula

$$f_{\mathrm{blob}}(\lambda_{0},\lambda_{1},\lambda_{2})=\{\begin{array}{ll} |\lambda_{2}|\frac{\left|\lambda_{2}\right|}{\left|\lambda_{0}\right|} & \text{if}\;\lambda_{2},\lambda_{1},\lambda_{0}<0,\\ 0 & \text{otherwise}, \end{array}$$ (3)

was proposed by Li et al.^8^ Such formulas produce high responses in the bright blob-like regions.

However, if some of the radial searches incorporate regions whose intensities are extremely high or low, huge intensity gradients of some specified directions are summed into the RST T(x), according to Eq. (1). The eigenvalues calculated in such regions may become $\lambda_{0}\ll \lambda_{1}\leq \lambda_{2}\leq 0$ or $\lambda_{1}\leq \lambda_{2}\leq 0\ll \lambda_{0}$, and the responses of an evaluation formula such as Eq. (3) become low.

**Fig. 2:**
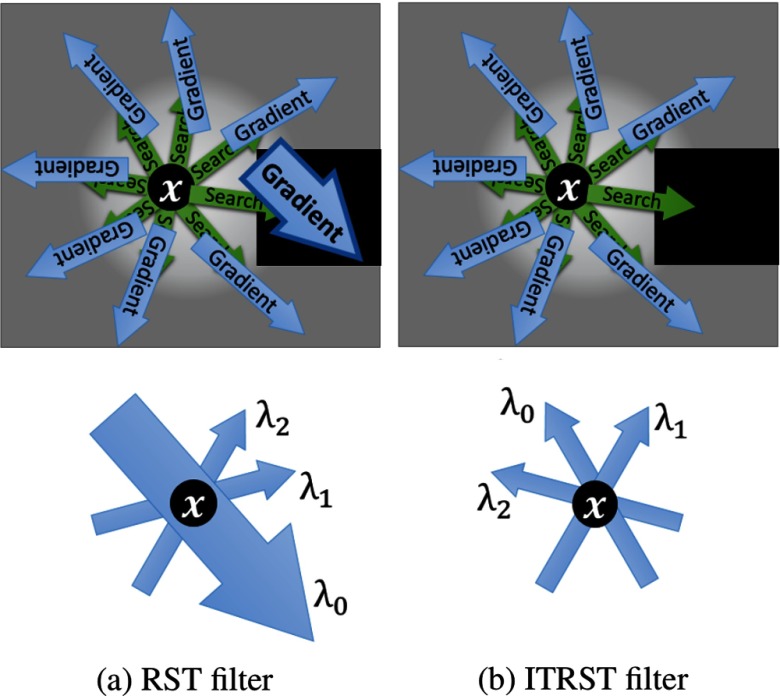
Schematic illustration showing differences between (a) RST and (b) ITRST filters. Point x is in sphere, and sphere is touching region with very low intensities. Top row represents intensity gradients that are summed into RST and ITRST, respectively. Bottom row represents magnitudes of eigenvalues $\lambda_{0}$, $\lambda_{1}$, $\lambda_{2}(|\lambda_{0}|\geq |\lambda_{1}|\geq |\lambda_{2}|)$ with corresponding eigenvectors of RST and ITRST, respectively.

To prevent the effect of the huge intensity gap explained above, we propose the ITRST filter. A schematic illustration showing the difference between the RST and the ITRST filters is summarized in Fig. 2. The ITRST filter introduces the prior knowledge of the target region to prevent summing huge intensity gradients into the ITRST. Intensity gradients at the location of higher or lower intensity than the thresholds are not summed into the ITRST. The ITRST is defined by modifying Eq. (1) as

$$T^{\prime }(x)=\underset{i}{\sum }\underset{j}{\sum }\alpha_{i,j}\gamma_{i,j}r_{i}g_{i,j}^{T},$$ (4)

where $\gamma_{i,j}$ is a function that classifies whether all points utilized for computing $g_{i,j}$ have the intensity within a predetermined range or not, which is defined as

$$\gamma_{i,j}=\{\begin{array}{ll} 1 & \text{if}\;t_{\text{dark}}\leq I(x^{\prime })\leq t_{\text{bright}}\;\text{for}\;\forall \;x^{\prime }\in N(i,j)\\ 0 & \text{otherwise}, \end{array},$$ (5)

where N(i,j) is a set of six-neighborhood voxels around $x_{i,j}$. In Eq. (5), $t_{\text{dark}}$ and $t_{\text{bright}}$ are upper and lower limits of target intensity range, respectively. The parameters $t_{\text{dark}}$ and $t_{\text{bright}}$ affect the enhancement results as follows. If we lower $t_{\text{dark}}$, this causes FPs of lymph nodes neighboring air regions. If we set $t_{\text{bright}}$ higher, this setting produces FPs of lymph nodes neighboring contrast-enhanced blood vessels (intensities of 150 to 300 H.U.). Choosing higher $t_{\text{dark}}$ or lower $t_{\text{bright}}$ makes the filter responses lower because it reduces the intensity gradients summed into the ITRST. When an accumulated opacity $\beta_{i}^{\prime }=\max(\sum_{j}\alpha_{i,j},\gamma_{i,j})≃1$ or a search length becomes $t_{\mathrm{len}}$ or larger, a search for the i’th search direction is terminated. Eigenvalues of $T^{\prime }(x)+T^{\prime T}(x)$, $\lambda_{0}^{\prime }$, $\lambda_{1}^{\prime }$, $\lambda_{2}^{\prime }(|\lambda_{0}^{\prime }|\geq |\lambda_{1}^{\prime }|\geq |\lambda_{2}^{\prime }|)$ can be utilized in the same manner as those of the RST filter.

### 2.2 Mediastinal Lymph Node Detection

#### 2.2.1 Overview

This section explains a mediastinal lymph node detection algorithm from CT volumes, which is based on the ITRST filter. In this algorithm, we assume that the input of the algorithm is a chest CT volume. The output is mediastinal lymph node detection results. Detection targets are the mediastinal lymph nodes with the specified least short axis or above. This is because enlarged lymph nodes have high possibility to be metastasized. The entire process consists of (1) preprocessing, (2) obtaining candidate regions by thresholding against the responses of the ITRST filter, and (3) FP reduction using machine learning.

The input volume I is a chest CT volume. The ground-truth binary volume $I_{g}$ of mediastinal lymph nodes is required if I is utilized for training only. The output is a binary volume $I_{\text{out}}$, which has lymph node regions denoted by the value 1. We describe the size of each lymph node using principal components analysis. We focus on the detection of lymph nodes above a specified size, defined by a short axis length of at least $r_{\text{target}}$ (mm).

#### 2.2.2 Preprocessing

First, we interpolate an input volume to generate an isotropic resolution volume. We apply the cubic interpolation on I to obtain a volume with isotropic resolution $w_{\mathrm{reso}}\;(\mathrm{mm})\times w_{\mathrm{reso}}\;(\mathrm{mm})\times w_{\mathrm{reso}}\;(\mathrm{mm})$ per voxel.^26^ Furthermore, we apply a Gaussian smoothing filter with standard deviation $\sigma_{\text{smooth}}$ (mm) for reducing noise and making local gradients more stable. We denote the preprocessed input volume as $I^{\prime }$.

#### 2.2.3 Target region of detection

We restrict the target region for lymph node detection to inside the mediastinal region, which we define as the area between the lungs. A lung region $A_{\text{lung}}$ is segmented by an automatic segmentation algorithm. We use a lung segmentation algorithm similar to Hu et al.^27^

First, we obtain air regions $A_{\text{air}}$ in $I^{\prime }$ as regions having lower intensities than a given threshold $t_{\text{air}}$ and not touching the boundary of the input CT volume. Then, we select the first and second largest connected components from the air regions detected. If the second largest component of the air regions is less than 20% of the largest one, we choose the largest component of the air regions as $A_{\text{lung}}$. Finally, the mediastinum region $A_{\text{media}}$ is obtained by $A_{\text{lung}}$ using Procedure 1. The function max(a) represents the maximum value of a in this procedure.

**Procedure 1:** Segmentation of mediastinum region $A_{\text{media}}$

Input: lung region $A_{\text{lung}}$
**for**z=0 to max(z)**do**
**for**y=0 to max(y)**do**
$a_{1}=0$
**for**x=0 to max(x)−1**do**
**if**$A_{\text{lung}}(x,y,z)=1\cap A_{\text{lung}}(x+1,y,z)=0$**then**
$a_{1}\leftarrow a_{1}+1$
**end if**
**end for**
**if**$a_{1}\geq 2$**then**
$a_{2}=0$
**for**x=0 to max(x)−1**do**
**if**$1\leq a_{2}<a_{1}\cap A_{\text{lung}}(x+1,y,z)=0$**then**
$A_{\text{media}}(x,y,z)\leftarrow 1$
**end if**
**if**$A_{\text{lung}}(x,y,z)=1\cap A_{\text{lung}}(x+1,y,z)=0$**then**
$a_{2}\leftarrow a_{2}+1$
**end if**
**end for**
**end if**
**end for**
**end for**
Output: mediastinum region $A_{\text{media}}$

We assume that the x-axis of the input volume I corresponds with the right-to-left direction of the body, the y-axis corresponds with the front-to-back direction, and z-axis corresponds with the head-to-foot direction. We scan the lung region $A_{\text{lung}}$ from each pair of (y,z) toward the right-to-left direction (along the x-axis). For each scan, we check whether no fewer than two components exist in the scan line. This means that there are right and left lung regions in the scan line. If no fewer than two components exist, we fill the gap between each component with the value 1.

#### 2.2.4 Initial lymph node detection using ITRST filter

We obtain the candidate regions using the ITRST filter. First, we apply the ITRST filter with the evaluation Eq. (3) to $I^{\prime }$. Since each point of $I^{\prime }$ has one response, we obtain the volume of filter response F. Because responses of the ITRST filter sometimes become zero in the blob-like regions such as an individual hole, we apply a median filter of $w_{\text{hole}}\times w_{\text{hole}}\times w_{\text{hole}}$ (voxels) to F to normalize such points. Then, we perform thresholding on the result of the median filter $F^{\prime }$ as

$$F_{\mathrm{blob}}^{\prime }(x)\geq t_{\mathrm{blob}},$$ (6)

with the threshold value $t_{\mathrm{blob}}$. Regions of connected components whose volumes are less than that of the sphere with radius $t_{\text{small}}$ (mm) and ones that are not touching the mediastinum region $A_{\text{media}}$ are eliminated. The remaining are denoted as candidate regions.

#### 2.2.5 FP reduction

The ITRST filter detects many FP regions as initial lymph node candidate regions. The SVM classifier is utilized to classify each candidate region into TP or FP regions. (10+7|D|)-dimensional features are utilized for each candidate region, as shown in Table 1. D is the permutation of radii utilized for computation of features related to the intensity. The number of elements of D is |D|, index of D is p(1≤p≤|D|), and one of its elements is written as d∈D. When d>0  mm, the target region for computing the intensity features is obtained by dilation of the candidate region, by using a structure element of a sphere whose radius is d  mm. The target region represents a neighbor of the candidate region, and larger values of d make the target region thicker. When d=0  mm, the target region is the same as the candidate region. Features are computed for both the training step and the testing step, and utilized as follows.

• Training step
  Feature vectors obtained from candidate regions of all volumes in the training dataset are used for training of the SVM.^22^ The set of lymph node regions whose short axis is at least $r_{\text{target}}w_{\text{train}}\;\mathrm{mm}$ in the ground truth $I_{g}$ is written as G. $r_{\text{target}}$ is a parameter representing the minimum length of the short axis of target lymph nodes. $w_{\text{train}}(0<w_{\text{train}}<1)$ is the parameter for setting the smallest size of a lymph node that is utilized for training. If the center of a candidate region is in one of the lymph node regions of G, its feature vector is utilized as a positive sample. If the center of a candidate region is outside of the lymph node regions, its feature vector is utilized as a negative sample.
• Testing step
  A candidate region extracted from a test volume is classified into TP or FP with its feature vector by using an SVM trained as above. If a candidate region is predicted as TP, the values of the output binary volume $I_{\text{out}}$ are set to 1 in the candidate region.

**Table 1:** Features for FP reduction step.

Group	Symbol	Definition
	$u_{1}$	Volume (${\mathrm{mm}}^{3}$)
	$u_{2}$	Surface area (${\mathrm{mm}}^{2}$)
Geometry	$u_{3}$	Sphericity
	$u_{4}$	Maximum length from contour (mm)
	$u_{5}$	Length of long axis (mm)
	$u_{6}$	Length of short axis (mm)
	$u_{7}$	x-coordinate
Location	$u_{8}$	Normalized y-coordinate of $C_{k}$ in bounding box of lung
	$u_{9}$	z-coordinate
	$u_{10+7(p-1)}$	Average
	$u_{11+7(p-1)}$	Variance
	$u_{12+7(p-1)}$	Median
Intensity	$u_{13+7(p-1)}$	Maximum of I in the target region. See Sec. 2.2.5 for details.
	$u_{14+7(p-1)}$	Minimum
	$u_{15+7(p-1)}$	Skewness
	$u_{16+7(p-1)}$	Kurtosis

## 3 Materials and Methods

### 3.1 ITRST Filter

#### 3.1.1 Materials

**Fig. 3:**
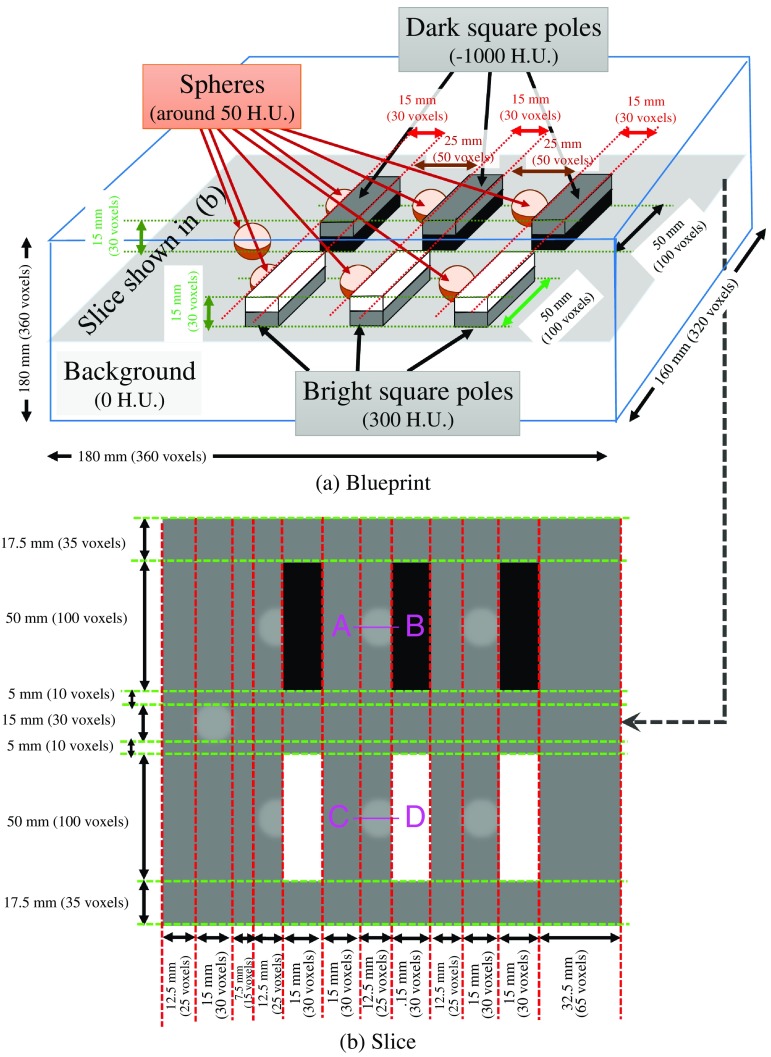
Synthetic examples of solid objects. (a) Blueprint of artificially generated volume. Slice contains centers of all spheres. (b) Slice containing one isolated sphere and six spheres touching bright (300 H.U.) or dark (−1000  H.U.) square poles.

An artificially generated volumetric image is used for evaluation of the ITRST filter. This volume includes seven objects imitating lymph nodes, three objects imitating contrast-enhanced blood vessels, and three objects imitating air regions. Figures 3(a) and 3(b) show the blueprint and one slice of the artificially generated volume containing the synthetic objects, respectively. This volume contains one isolated sphere, three spheres overlapping with 300 H.U. square poles, and three spheres overlapping with −1000  H.U. square poles. The background of the volume is 0 H.U. First, the spheres with a diameter of 15 mm are drawn as uniform of 50 H.U., and Gaussian smoothing of σ=1.0  mm is applied to make the spheres similar to lymph nodes of real CT volumes. After that square poles with thickness of 15 mm and length of 50 mm are drawn.

#### 3.1.2 Filter response evaluation

We apply the ITRST, RST, and Hessian filters to the artificially generated volume explained above. First, we examine the filter responses. In this experiment, we obtain the eigenvalue profile on the two spheres shown in Fig. 3(b). The line A–B in Fig. 3(b) crosses one of the spheres overlapping a dark region. The line C–D is one of the spheres overlapping a bright region.

Parameters used in the experiments are shown in Table 4(a). We set $t_{\text{bright}}=100\;\mathrm{H.U.}$ and $t_{\text{dark}}=-100\;\mathrm{H.U.}$ since the spheres have comparability with lymph nodes, which range from −100 to 100 H.U. We also set $t_{\mathrm{len}}=15\;\mathrm{mm}$ as the upper limit of the radius of lymph nodes to be detected in real CT volumes.

### 3.2 Mediastinal Lymph Node Detection

#### 3.2.1 Materials

Forty-seven chest CT volumes were prepared for the experiments on mediastinal lymph node detection. These volumes were authorized by the ethics committee of Nagoya University Hospital. The specifications of the volumes are shown in Table 2. We evaluate the performance of lymph nodes for a range of minimum sizes: the short axis is at least $r_{\text{target}}\in \{10,7.5,5\;\mathrm{mm}\}$. Ground-truth data are a set of mediastinal lymph node labels. Two technical researchers who have sufficient knowledge of lymph nodes first manually traced lymph node candidate regions on the CT volumes. Then, an expert radiologist confirmed these traced data including missing lymph nodes on CT slices. Table 3 shows the number of lymph nodes of each size category.

**Table 2:** Specification of CT volumes used in experiments on mediastinal lymph node detection.

Item	Spec
Number of volumes	47
Dimension	3
Phase	Arterial
Device	Aquilion 64, Toshiba
Reconstruction function	FC11
Size	512×512×(338–463) voxels
Resolution	$(0.625-0.782)\times (0.625-0.782)\times (0.799-0.801)\;{\mathrm{mm}}^{3}$

**Table 3:** Number of lymph nodes categorized by short axes.

Size category	Number of lymph nodes
[10 mm, ∞)	57
[7.5 mm, 10 mm)	62
[5 mm, 7.5 mm)	145
[3 mm, 5 mm)	284
Total	548

#### 3.2.2 Initial detection performance evaluation

We compute FROC curves of initial detection results by changing the parameter $t_{\mathrm{blob}}$, for comparing the ITRST, RST, and Hessian filters. The filter output is binarized at different thresholds ($t_{\mathrm{blob}}=20$, 40, 80, and 160 for the ITRST filter, $t_{\mathrm{blob}}=20$, 40, 80, and 160 for the RST filter, and $t_{\mathrm{blob}}=2000$, 4000, 8000, and 16,000 for the Hessian filter), as explained in Sec. 2.2.4.

Each point on FROC curves represents the averages of the detection rate and the number of FPs/volume among all volumes. The corresponding error bars represent the standard deviation of the detection rate. Our detection targets are mediastinal lymph nodes whose short axes are at least $r_{\text{target}}$ (mm). Each mediastinal lymph node is classified and counted as TP or FN. If at least one region produced by the filter overlaps with a mediastinal lymph node of the detection target, the lymph node is counted as TP. The detection rate of each volume is defined as

$$(\text{Detection rate of each volume})=\frac{\left(\text{Number of}\;\mathrm{TPs}\right)}{\left(\text{Number}\;\mathrm{of}\;\text{detection}\;\text{targets}\right)}.$$

The number of FPs in each volume is the count of regions produced by the filter that do not overlap with any lymph nodes or lung cancers.

The FROC curves are drawn for each value (5, 7.5, and 10 mm) of the least short axis parameter $r_{\text{target}}$. Parameters of $t_{\text{bright}}$, $t_{\text{dark}}$, and $t_{\mathrm{len}}$ are the same as the experiment of Sec. 3.1. Other parameters are set empirically [Table 4(b)]. The air region segmentation threshold $t_{\text{air}}$ is set as −200  H.U. This threshold is set to be sufficiently lower than lymph nodes or surrounding soft tissues, which have around −100  H.U. or above. Isotropic resolution parameter $w_{\mathrm{reso}}$ is set as 0.625 mm. This equals the smallest pixel size of axial slices of the CT volumes (Table 2). Standard deviation of Gaussian smoothing filter $\sigma_{\text{smooth}}$ is set as 1 mm. This setting is good for reducing noise on chest CT volumes without severely blurring edges. The parameter $w_{\text{hole}}$, the size of median filter applied to the output of the ITRST filter, is set as three voxels. This is the smallest size of median filter.

**Table 4:** Constant parameter values. (a) ITRST filter. (b) Mediastinal lymph node detection: initial detection evaluation. (c) Mediastinal lymph node detection: overall detection performance evaluation. Note that $r_{\text{target}}$, $t_{\mathrm{blob}}$, and $w_{F}$ are not shown here since they are varied for evaluating the performance in different conditions.

Symbol	Value	Definition
(a) ITRST filter		
$t_{\text{bright}}$	100 H.U.	Upper limit of intensity target range of ITRST filter
$t_{\text{dark}}$	−100 H.U.	Lower limit of intensity target range of ITRST filter
$t_{\mathrm{len}}$	15 mm	Maximum search length of ITRST filter
(b) Mediastinal lymph node detection: Initial detection evaluation		
$t_{\text{bright}}$	100 H.U.	Upper limit of intensity target range of ITRST filter
$t_{\text{dark}}$	−100 H.U.	Lower limit of intensity target range of ITRST filter
$t_{\mathrm{len}}$	15 mm	Maximum search length of ITRST filter
$t_{\text{air}}$	−200 H.U.	Threshold for dividing air and other regions
$w_{\mathrm{reso}}$	0.625 mm	Isotropic resolution at which input volume is interpolated
$\sigma_{\text{smooth}}$	1 mm	Standard deviation of Gaussian smoothing filter
$w_{\text{hole}}$	3 voxels	Size of median filter applied for output of ITRST filter
(c) Mediastinal lymph node detection: Overall detection performance evaluation		
$w_{\text{train}}$	0.5	Tolerance for using feature vectors extracted from candidate regions of smaller lymph nodes than $r_{\text{target}}$ for training SVM
D	{0,1,2} mm	Width of regions for computing feature values regarding intensity

#### 3.2.3 Overall detection performance evaluation

We compute the FROC curves as the overall performance. This performance includes FP reduction by SVM. An FROC curve is obtained by changing the weighting parameter $w_{F}$ of negative samples for the SVM classifier^22^ utilized in the FP reduction step (explained in Sec. 2.2.5). Leave-one-out cross validation is conducted to evaluate the performance of FP reduction for each volume. The SVM classifier is tested with the data not used in the training process.

We also conduct a statistical test (Fisher’s exact test) of the detection rate obtained by the ITRST filter and the others. For fair comparison of detection rate between the filters, we draw the FROC curve with various values of $w_{F}$ and estimate the detection rate at the point of 10.0 FPs/volume on the FROC curve.

The threshold for the filter output is chosen as $t_{\mathrm{blob}}=20$ for the ITRST and RST filters, and $t_{\mathrm{blob}}=2000$ for the Hessian filter, since these settings of $t_{\mathrm{blob}}$ gave the highest initial detection rate with each filter. To compute the FROC curves, the weighting parameter $w_{F}$ is changed as 0.025, 0.05, 0.075, 0.10, 0.125, 0.15, 0.20, 0.25, 0.30, 0.40, and 0.50. Other parameters utilized for the FP reduction step are set empirically [Table 4(c)]. The parameter $w_{\text{train}}$ for tolerance of using feature vectors extracted from candidate regions of lymph nodes smaller than the target for training SVM is set as 0.5 for preventing false negatives of lymph nodes whose short axis is almost the same as $r_{\text{target}}$. Permutation D representing the width of regions for computing feature values regarding intensity is set as {0,1,2}mm for focusing on the inside and neighboring regions of each candidate region. The LIBSVM 3.17^28^ library is utilized as an SVM implementation.

## 4 Results

### 4.1 ITRST Filter

**Fig. 4:**
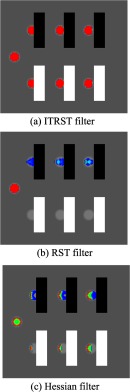
Responses for artificially generated volume. (a) ITRST filter. (b) RST filter. (c) Hessian filter. Color scheme is same as (a). Blue represents low response (around 1), yellow represents medium response (around 125), and red represents high response (around 250).

The responses of the ITRST, RST, and Hessian filters for the artificially generated volume are shown in Figs. 4(a)–4(c), respectively. The responses were higher in most of the sphere regions than those of the RST and Hessian filters, despite the overlapping of square poles.

**Fig. 5:**
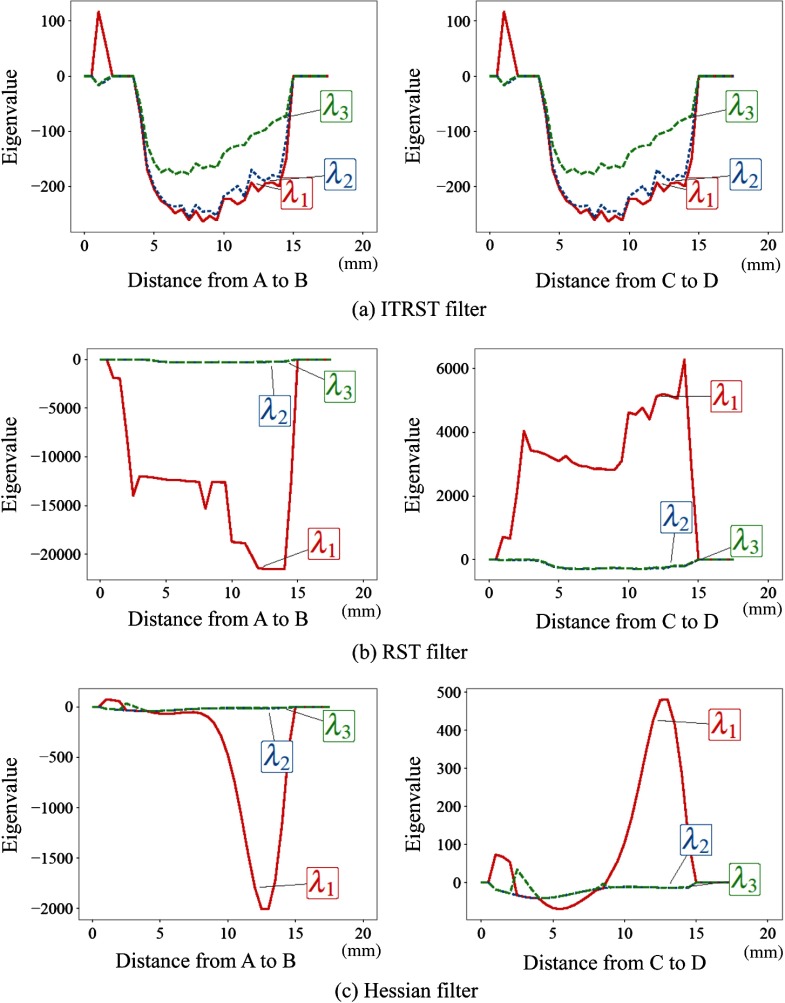
Eigenvalue profiles on lines A–B and C–D shown in Fig. 3(b). (a) ITRST filter. (b) RST filter. (c) Hessian filter.

The eigenvalue profiles obtained by the ITRST, RST, and Hessian filters are shown in Figs. 5(a)–5(c), respectively. By using the ITRST filter, all eigenvalues were negative, and their magnitudes do not differ much in the entire part of both spheres. By using the RST filter, $\lambda_{1}$ became far smaller or larger than other eigenvalues in the spheres touching the bright and dark square poles, respectively. Eigenvalues of the Hessian filter also became positive in the parts near the square poles.

### 4.2 Mediastinal Lymph Node Detection

#### 4.2.1 Initial detection evaluation

**Fig. 6:**
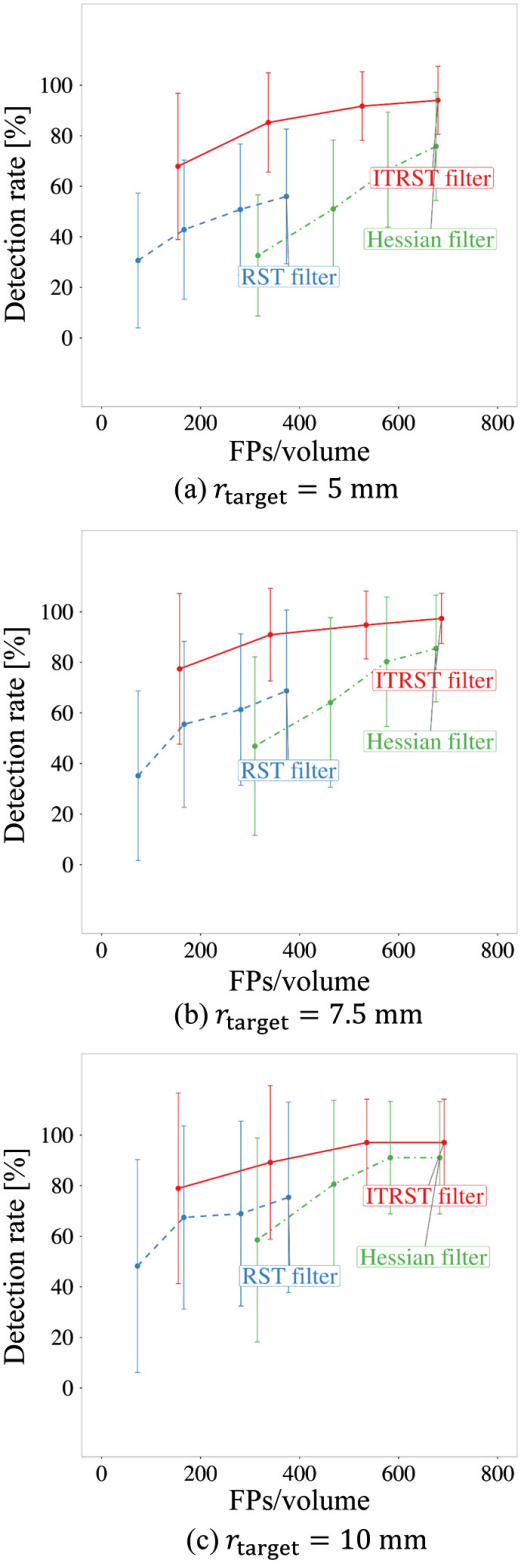
FROC curves obtained after initial detection ($t_{\mathrm{blob}}$ for 20.0, 40.0, 80.0, and 160.0 for ITRST and RST filters, and $t_{\mathrm{blob}}$ for 2000, 4000, 8000, and 16,000 for Hessian filter). (a) $r_{\text{target}}=5\;\mathrm{mm}$. (b) $r_{\text{target}}=7.5\;\mathrm{mm}$. (c) $r_{\text{target}}=10\;\mathrm{mm}$.

The FROC curves of initial detection are shown in Fig. 6. As shown in Table 5, a higher detection rate was achieved by the proposed algorithm (ITRST filter) than by the RST filter. For instance, when $r_{\text{target}}=10\;\mathrm{mm}$ and $t_{\mathrm{blob}}=20$, 97.1% of lymph nodes were detected with 692.1 FPs/volume by the proposed algorithm (ITRST filter). Using the RST ($t_{\mathrm{blob}}=20$) or Hessian filters ($t_{\mathrm{blob}}=2000$), 75.4% or 91.1% were detected with 377.8 or 683.2 FPs/volume, respectively. Examples of the detection results are shown in Fig. 8.

**Table 5:** Comparison of lymph node detection performances. Note that symbol * represents performance shown in publication. Their experiments were performed using different datasets or criteria from ours.

Algorithm	Least short axis (mm)	Initial detection		After FP reduction	
		Detection rate (%)	FPs/volume	Detection rate (%)	FPs/volume
Proposed algorithm	5	94.0±13.4	679.6±83.6	68.4±25.7	10.0±5.2
RST filter	5	56.0±26.7	373.5±80.7	44.0±24.6	6.6±4.2
Hessian filter	5	75.8±21.4	675.8±117.0	54.9±25.6	11.9±5.3
Proposed algorithm	7.5	97.4±9.9	686.8±84.3	72.8±29.6	10.1±5.3
RST filter	7.5	68.7±32.0	373.7±80.4	55.8±33.4	6.6±4.3
Hessian filter	7.5	85.5±21.1	675.6±118.1	68.6±33.5	11.7±5.3
Proposed algorithm	10	97.1±17.1	692.1±82.6	84.2±31.0	9.1±5.1
RST filter	10	75.4±37.6	377.8±77.2	63.9±41.4	6.3±4.2
Hessian filter	10	91.1±22.2	683.2±121.7	78.2±35.3	11.1±5.1
*Roth et al.^23^	10	—	—	84	6
*Liu et al.^29^	10	—	—	80	8
*Feulner et al.^11^	10	—	—	60.9	6.1

#### 4.2.2 Overall detection performance evaluation

**Fig. 7:**
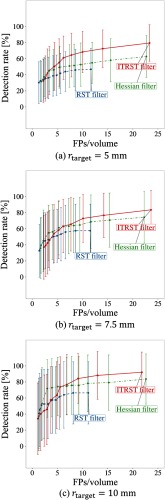
FROC curves obtained after FP reduction ($w_{F}$ for 0.025, 0.05, 0.075, 0.10, 0.125, 0.15, 0.20, 0.25, 0.30, 0.40, and 0.50) with $t_{\mathrm{blob}}=20$ for ITRST and RST filters, and $t_{\mathrm{blob}}=2000$ for Hessian filter. (a) $r_{\text{target}}=5\;\mathrm{mm}$. (b) $r_{\text{target}}=7.5\;\mathrm{mm}$. (c) $r_{\text{target}}=10\;\mathrm{mm}$.

Table 5 and Fig. 7 show the overall performance calculated from the output of the mediastinal lymph node detection algorithm, with parameters $t_{\mathrm{blob}}=20$ ($t_{\mathrm{blob}}=2000$ for Hessian filters) and $w_{F}=0.075$. For example, when $r_{\text{target}}=10\;\mathrm{mm}$, 84.2% of lymph nodes were detected with 9.1 FPs/volume by the proposed algorithm (ITRST filter). Table 6 displays the results of Fisher’s exact test at 10.0 FPs/volume. Performance of the proposed algorithm was not always significantly better. It was shown that detection rates of ITRST and RST filters were significantly different (p<0.05) with all settings (5, 7.5, and 10 mm) of the least short axis. On the other hand, detection rates of the ITRST and Hessian filters were significantly different when the least short axis was 5 mm.

**Table 6:** Fisher’s exact test among detection rate at 10.0 FPs/volume.

Least short axis (mm)	Detection rate (%)			p-value	
	ITRST	RST	Hessian	ITRST-RST	ITRST-Hessian
5	68.3	46.4	53.0	0.003	0.043
7.5	72.5	57.5	65.6	0.037	0.357
10	85.1	66.4	76.8	0.003	0.207

## 5 Discussion

### 5.1 ITRST Filter

It is clear that responses of the ITRST filter were higher in most of the sphere regions than those of the RST filter, even if the square poles were overlapping, as shown in Fig. 4. It suppressed the negative effect of the regions with much higher or lower intensity than the detection target. The Hessian filter yielded positive values only on some parts of the spheres.

The magnitude of $\lambda_{1}$ computed from the ITRST filter was similar to $\lambda_{2}$ and $\lambda_{3}$, and it was negative on almost all of A–B or C–D, as shown in Fig. 5(a). Huge intensity gradients were not summed into the ITRST, and the eigenvalues followed the condition of the bright blob-like structure. In contrast, as shown in Fig. 5(b), the magnitude of $\lambda_{1}$ computed from the RST filter was far larger than $\lambda_{2}$ and $\lambda_{3}$ in the sphere. This is because huge intensity gradients directed from the sphere to the square pole were accumulated into the RST. Figure 5(c) shows the magnitude of $\lambda_{1}$ computed from the Hessian filter, which becomes large in the part near the square poles in the Hessian filter. The eigenvalues did not follow the condition of the bright blob-like structure ($\lambda_{1}≃\lambda_{2}≃\lambda_{3}\ll 0$) in the part with large magnitude of $\lambda_{1}$, and the responses became low according to Eq. (3).

By comparing the FROC curves of initial detection shown in Fig. 6, it is clear that the ITRST filter had a higher detection rate for both large and small lymph nodes than the RST filter. Results after FP reduction of the proposed algorithm (ITRST filter) were also better than those of the RST filter. The ITRST filter is more useful than the RST filter for mediastinal lymph node detection.

### 5.2 Mediastinal Lymph Node Detection

#### 5.2.1 Efficacy of ITRST filter

**Fig. 8:**
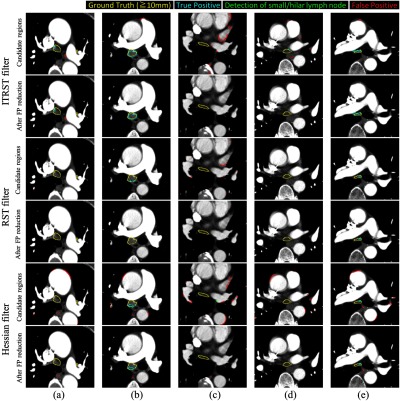
Examples of detection results (a)–(e) with parameters $t_{\mathrm{blob}}=20$ and $r_{\text{target}}=10\;\mathrm{mm}$. Yellow denotes ground truth. Cyan denotes TP detection. Red denotes FP detection. Green represents detection of small or hilar lymph nodes. First two rows represent candidate regions and after FP reduction using ITRST filter. Third and fourth rows represent results of RST filter. Fifth and sixth rows are of Hessian filter.

The lymph node shown in Fig. 8(a) was properly detected by the proposed algorithm (ITRST filter) and the Hessian filter, while the RST filter was not able to detect it. This was likely due to the presence of contrast-enhanced blood vessels and the air region adjacent to the lymph node. In contrast to the RST filter, the ITRST filter reduced the impact of the large intensity gradients around the lymph node so that the eigenvalues still followed the condition of the bright blob-like structure. This lymph node was not surrounded entirely by extremely high or low intensity regions, so some intensity gradients derived from soft tissue could still be utilized to descibe the bright blob-like structure.

The lymph node shown in Fig. 8(b) was detected initially by all algorithms. The candidate region obtained by the RST filter was poorly segmented because of the negative effect of the contrast-enhanced blood vessels and the air, and it was removed by SVM. However, the candidate region obtained by the ITRST filter covered most of the lymph node region, and it was classified as a lymph node. The ITRST filter prevented the negative effect of surrounding regions and contributed to accurate classification. Although the candidate region obtained by the Hessian filter was a little smaller than that of the ITRST filter, it was also properly classified.

#### 5.2.2 False negatives still produced

Some lymph nodes were still missed by the ITRST filter, as shown in the FROC curves in Fig. 6. This is because they tend to be sandwiched by extremely high or low intensity regions. For instance, the lymph node shown in Fig. 8(c) could not be detected by the ITRST or the RST filters. It was sandwiched by the contrast-enhanced blood vessels and the air region, and a very small amount of soft tissue was touching the lymph node. Most gradient vectors around it were not summed into the ITRST, so the magnitude of eigenvectors did not follow the condition of the bright blob-like structure. In contrast to the ITRST or RST filters, the Hessian filter did not strongly suffer from intensity differences between lymph nodes and neighboring regions. The small candidate region detected by the Hessian filter was finally classified into lymph node class after the FP reduction process. It remains as future work to improve the ITRST filter such that it can segment lymph nodes surrounded by extremely high or low intensity regions.

The lymph node shown in Fig. 8(d) was initially detected by the ITRST filter, but it was removed by the SVM classifier. To prevent generating such false negatives, we will improve the classification accuracy by introducing deep learning techniques in future work. Note that the candidate region of the ITRST filter was properly classified as a lymph node with $w_{F}=0.025$.

#### 5.2.3 Promise for application to segmentation

As shown in Fig. 8(e), some lymph nodes are detected by all filters (ITRST, RST, and Hessian). However, the ITRST filter produced more proper segmentation results of lymph nodes than other filters. In the future, the ITRST filter can be improved for application to segmentation of lymph nodes, not only for detection. This will assist radiologists in measuring the size and shape of each lymph node.

### 5.3 Lung Area Segmentation

**Fig. 9:**
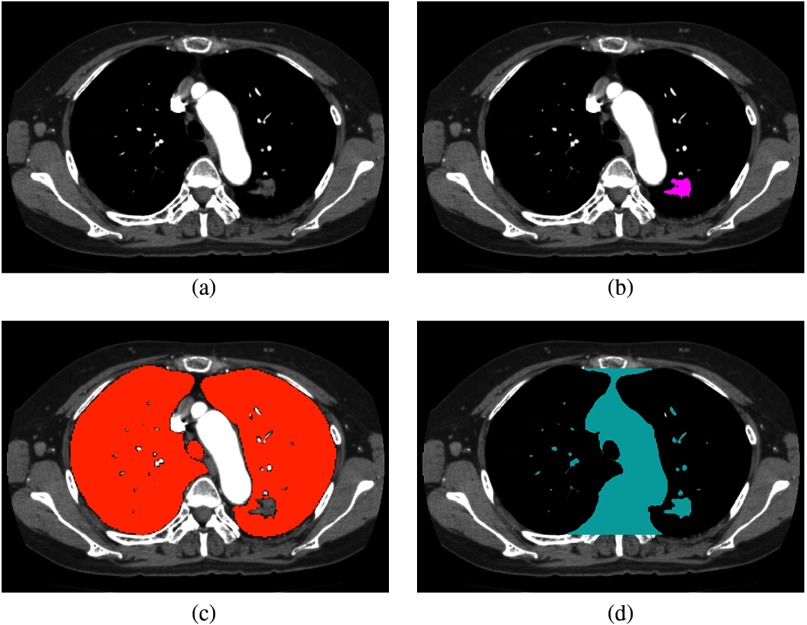
Target region extraction on volume with lung cancer region. (a) Input volume. (b) Lung cancer region. (c) Lung region. (d) Target region.

We have extracted mediastinum regions from CT volumes by extracting lung regions. There is some possibility to fail in lung region segmentation in a pathological lung with cancer, as shown in Fig. 9. However, that does not affect the subsequent processes since lung segmentation is only for obtaining the mediastinum region sandwiched between the lungs. The lung cancer region is merged into the target region.

## 6 Conclusions

In this paper, we proposed a mediastinal lymph node algorithm based on the ITRST filter. The conventional RST filter cannot detect some lymph nodes because of neighboring regions of lymph nodes, which have extremely high or low intensities. We proposed the ITRST filter by modifying the RST filter to prevent such negative effects by introducing knowledge about the intensity range of the detection target. It allows us to exclude neighboring regions for computing the filter response and increase the detection rate of lymph nodes.

We evaluated the efficacy of the ITRST filter by applying it to both an artificially generated volume and chest CT volumes. In an experiment on synthetic data, the ITRST filter produced high responses in the spheres neighboring bright or dark square poles, while responses of the RST filter were very low or zero. These results showed that the ITRST filter can prevent the negative effect caused by such neighboring regions, in contrast to the RST filter.

Furthermore, experimentation with real clinical images for mediastinal lymph node detection showed that the ITRST filter outperformed the RST filter. This is because most of the mediastinal lymph nodes adjacent to air or contrast-enhanced blood vessels in the chest CT volumes can be detected using the ITRST filter. The detection performance after FP reduction is also better than the RST filter. The proposed ITRST filter could potentially be used for detection of other organs or tissues of interest in medical imaging.
